# EDTA-dependent pseudothrombocytopenia mimicking dengue-associated severe thrombocytopenia: a case report

**DOI:** 10.1097/MS9.0000000000003916

**Published:** 2025-09-15

**Authors:** Osama Mohamed Abdulsalam Ali, Mohamed Essam Abdulaziz Badawy, Khalid Osman Mohamed

**Affiliations:** a Internal Medicine Department, Mohammad Dossary Hospital, Al-Khobar, Saudi Arabia; b Faculty of Medicine, University of Bahri, Khartoum, Sudan

**Keywords:** dengue fever, EDTA-dependent pseudothrombocytopenia, peripheral blood smear, platelet clumping, thrombocytopenia, thrombocytopenia

## Abstract

**Introduction:**

We report a case of suspected dengue fever that was ultimately diagnosed as ethylenediaminetetraacetic acid (EDTA)-dependent pseudothrombocytopenia in a 24-year-old man presenting with frontal headache, malaise, and recent fever.

**Case presentation:**

A previously healthy 24-year-old man presented with thrombocytopenia, with an initial platelet count of 25 × 10^9^/L. He reported a 1-week history of frontal headache, severe dizziness, generalized weakness, and recent fever. There was no history of bleeding or bruising. Given the degree of thrombocytopenia and a regional increase in dengue cases, dengue fever was the provisional diagnosis. The patient was admitted for further evaluation and conservative management. A peripheral blood smear revealed morphologically normal platelets with marked clumping, raising suspicion of EDTA-dependent pseudothrombocytopenia. Paired blood samples were drawn in EDTA and sodium citrate tubes. The EDTA sample showed a platelet count of 42 × 10^9^/L, whereas the citrate sample showed a count of 191 × 10^9^/L, confirming the diagnosis. The patient remained clinically stable and was discharged. After discharge dengue serology returned back negative.

**Clinical discussion:**

In patients presenting with thrombocytopenia, the clinical picture is essential in guiding appropriate management. While it is important to maintain a low threshold for suspecting dengue fever, clinicians should also broaden the differential diagnosis to include pseudothrombocytopenia.

**Conclusion:**

This case report serves as a learning example that physicians should remain vigilant for EDTA-dependent pseudothrombocytopenia and request peripheral smears and citrate-based counts in cases of unexplained thrombocytopenia. Awareness of laboratory limitations in addition to correlation with clinical picture is a must for clinicians.

## Introduction

Pseudothrombocytopenia, also known as spurious thrombocytopenia, is a relatively uncommon *in vitro* phenomenon that occurs during specimen collection. It typically arises when blood is collected in a vial containing an anticoagulant, leading to platelet clumping and consequently resulting in falsely low platelet counts. In such cases, automated hematology analyzers are unable to provide an accurate platelet count. This condition most frequently occurs in blood samples anticoagulated with ethylenediaminetetraacetic acid (EDTA); however, it has also been reported with other anticoagulants such as citrate, heparin, and oxalate^[1,2]^.

**HIGHLIGHTS**

- Ethylenediaminetetraacetic acid (EDTA)-dependent pseudothrombocytopenia (EDTA-PTCP) is a laboratory artifact that can mimic severe thrombocytopenia.
- Misdiagnosis can lead to unnecessary and potentially harmful interventions, such as platelet transfusion.
- Peripheral blood smear examination is essential to identify platelet clumping.
- Repeat platelet count using non-EDTA anticoagulant confirms the diagnosis.
- Awareness of EDTA-PTCP can prevent clinical mismanagement and reduce financial burden.

Pseudothrombocytopenia can typically be identified by examining a peripheral blood smear, using an alternative anticoagulant to EDTA for blood collection, or by maintaining the sample at approximately 37°C prior to analysis[3].

This condition was first reported by Gowland *et al* in 1969[4]. Its prevalence has been estimated to range between 0.1% and 2% among hospitalized patients, with potentially higher rates in the outpatient population[5].

Recognizing EDTA-dependent pseudothrombocytopenia (EDTA-PTCP) is crucial to avoid unnecessary diagnostic procedures and interventions, such as bone marrow biopsy, surgical procedures, splenectomy, corticosteroid therapy, and platelet transfusions, which may be initiated under the mistaken assumption that the patient has true thrombocytopenia[2].

In this report, we present a case of EDTA-PTCP mimicking dengue fever in a 24-year-old man who was presented to our hospital with persistent thrombocytopenia and dengue fever symptoms. This case report has been reported in line with the SCARE Criteria[6].

## Case presentation

A 24-year-old man was referred to the emergency department from a primary care center due to a significantly low platelet count of 25 × 10^9^/L, along with a clinical suspicion of dengue fever. The referral was initiated based on his complaints of frontal headache, severe dizziness, and generalized weakness persisting for 1 week – symptoms that raised concern due to the potential risk of bleeding. He also reported a recent episode of fever but denied any history of bleeding, bruising, or other focal symptoms. His medical history was unremarkable, with no recent infections, hospitalizations, or hematologic disorders.

He was not taking any regular medications and had no recent antibiotic, antimalarial, or over-the-counter drug use. No medications had been recently discontinued. There were no known drug allergies or adverse drug reactions. No contraindications to restarting any regular medicines were identified.

There was no history of bleeding disorders, thrombocytopenia, autoimmune conditions, or other hematological diseases among first-degree relatives. His parents and siblings are healthy.

The patient is a non-smoker and denies alcohol consumption or recreational drug use. He lives independently, is able to perform activities of daily living without assistance, and drives regularly. He resides in an urban apartment with good access to healthcare.

No complaints of visual disturbance, photophobia, hearing loss, chest pain, palpitations, shortness of breath, abdominal pain, joint pain, or rash. No recent weight loss or night sweats.

Given the severity of the thrombocytopenia and the systemic symptoms in the context of a regional increase in dengue cases, dengue fever was considered the primary differential diagnosis. Based on these findings, the patient was admitted for further evaluation and management.

Upon admission, the patient was hemodynamically stable. His temperature ranged from 36.5°C to 36.6°C, respiratory rate was 20 breaths per minute, pulse varied between 62 and 88 beats per minute, and blood pressure ranged from 110/70 mmHg to 130/90 mmHg. His oxygen saturation on room air was between 94% and 98%, and random blood glucose levels were within normal limits.

A repeat complete blood count (CBC) was ordered, along with a peripheral blood smear and a coagulation profile that included International Normalized Ratio (INR), Prothrombin Time (PT), and Partial Thromboplastin Time (PTT). Serologic testing for dengue IgG and IgM antibodies, as well as malaria testing through both peripheral smear and a rapid diagnostic test, were also requested. The patient was started on intravenous fluids and empirical antibiotics. Blood grouping and crossmatching were performed, and platelet units were prepared in anticipation of a possible transfusion.

During the first day of admission, follow-up laboratory tests revealed a platelet count of 40 × 10^9^/L, a white blood cell count of 8.40 × 10^9^/L, and hemoglobin of 16.3 g/dL as detailed in Table 1.

**Table 1:** Complete blood count report

Parameter	Result	Unit	Reference range
Hemoglobin	16.3	g/dL	13–17.5
HCT	47.3	%	40–52
RBC	5.69	×10⁶/µL	4.5–5.9
MCV	83.1	fL	80–100
MCH	28.6	pg	27–33
MCHC	34.5	g/dL	31–37
RDW	12.0	%	11–15
Platelet count	40*	×10^3^/µL	150–450
Total WBC	8.40	×10^3^/µL	4–11
Neutrophil count	4.51	×10^3^/µL	2–7
Immature granulocyte count	0.02	×10^3^/µL	0.4
Lymphocyte count	2.77	×10^3^/µL	1–4.2
Monocyte count	0.80	×10^3^/µL	0.2–1
Eosinophil count	0.27	×10^3^/µL	0.2–0.5
Basophil count	0.05	×10^3^/µL	0.2
Neutrophil %	53.7	%	-
Lymphocyte %	33.0	%	-
Monocyte %	9.5	%	-
Eosinophil %	3.2	%	-
Basophil %	0.6	%	-

HCT, hematocrit; RBC, red blood cell count; MCV, mean corpuscular volume; MCH, mean corpuscular hemoglobin; MCHC, mean corpuscular hemoglobin concentration; RDW, red cell distribution width; WBC, white blood cell count.

A notification was sent to the Ministry of Health (MOH), and the infection control team was informed. Later that day, inflammatory markers were received and found to be within normal limits. C-reactive protein was 0.5 mg/dL, erythrocyte sedimentation rate was 7 mm/h, and lactate dehydrogenase was 190 U/L. Malaria testing was negative. These results are shown in Table 2.

**Table 2:** Inflammatory markers result

Parameter	Result	Unit	Reference range
C-reactive protein	0.5	mg/dL	1
Erythrocyte sedimentation rate	7	mm/h	2–10
Lactate dehydrogenase	190	U/L	120–246

Although the patient remained clinically stable, standard dengue precautions were implemented as a safety measure. On the following day, the head of the laboratory informed the medical team that the peripheral blood smear revealed excessive platelet clumping with morphologically normal platelets. In light of this finding, EDTA-dependent pseudothrombocytopenia was suspected. The laboratory recommended obtaining two simultaneous blood samples – one in an EDTA tube and another in a sodium citrate tube. The EDTA sample showed a platelet count of 42 × 10^9^/L, while the citrate sample revealed a significantly higher count of 191 × 10^9^/L. This confirmed the diagnosis of EDTA-dependent pseudothrombocytopenia. Other CBC parameters were also compared and are summarized in Table 3.

**Table 3:** Comparison of CBC parameters between EDTA and citrate samples

Parameter	EDTA sample	Citrate sample	Unit	Reference range
Hemoglobin	16.10	16.80	g/dL	13–17.5
HCT	45.40	47.40	%	40–52
RBC	5.52	5.78	×10^6^/µL	4.5–5.9
MCV	82.20	82.00	fL	80–100
MCH	29.60	29.10	pg	27–33
MCHC	35.5	35.40	g/dL	31–37
RDW	11.90	11.90	%	11–15
Platelet count	**42.0**	**157.0**	×10^3^/µL	150–450
Total WBC	6.15	5.69	×10^3^/µL	4–11
Neutrophil Count	3.47	3.37	×10^3^/µL	2–7
Immature Granulocyte count	0.01	0.02	×10^3^/µL	0.4
Lymphocyte count	1.73	1.79	×10^3^/µL	1–4.2
Monocyte count	0.75	0.59	×10^3^/µL	0.2–1
Eosinophil count	0.16	0.16	×10^3^/µL	0.2–0.5
Basophil count	0.04	0.05	×10^3^/µL	0.2
Neutrophil %	56.4	56.6	%	-
Lymphocyte %	28.1	30.0	%	-
Monocyte %	12.2	9.9	%	-
Eosinophil %	2.6	2.7	%	-
Basophil %	0.7	0.8	%	-

CBC, complete blood count; EDTA, ethylenediaminetetraacetic acid; HCT, hematocrit; RBC, red blood cell count; MCV, mean corpuscular volume; MCH, mean corpuscular hemoglobin; MCHC, mean corpuscular hemoglobin concentration; RDW, red cell distribution width; WBC, white blood cell count.

Bold represents the difference between initial (EDTA sample) and Citrate sample.

Dengue serology results later returned as non-reactive, with an IgG level of 6.89 and an IgM level of 3.46.

## Follow-up and outcomes

The patient remained clinically stable and asymptomatic throughout his hospital stay. After 2 days of observation, he was discharged in good condition. He was counseled about the benign nature of his condition and advised to inform future healthcare providers of his EDTA sensitivity to avoid misdiagnosis and prevent unnecessary interventions.

## Discussion

Thrombocytopenia commonly associated with dengue fever, and patients often present at varying levels of severity. Platelet counts typically remain low for approximately 5 days, followed by a recovery phase that usually leads to normalization by the 10th day after fever onset[7]. However, not all cases of thrombocytopenia in febrile patients are attributable to dengue fever.

EDTA-PTCP is a laboratory artifact caused by antiplatelet autoantibodies that lead to platelet aggregation in EDTA-anticoagulated blood, which can be misinterpreted for true thrombocytopenia by automated analyzers[8]. This can be identified through examination of a peripheral blood smear and an abnormal platelet histogram, often demonstrating the characteristic “sawtooth” pattern[9].

Previous studies have shown that EDTA can chelate calcium ions (Ca^2^⁺), leading to the dissociation of the calcium-dependent platelet membrane glycoprotein IIb/IIIa complex, which promotes *in vitro* platelet clumping^[5,10–12]^. The exposure of epitopes on GPIIb/IIIa is recognized by circulating antiplatelet autoantibodies, typically of the IgG or IgM class and less commonly IgA, triggering platelet clumping, and this agglutination tends to occur more easily at lower temperatures than at 37°C^[8,13]^.

In Saudi Arabia, where cases of dengue fever have been increasingly reported[14], the MOH has consistently emphasized the importance of early detection, issuing frequent alerts regarding suspected cases. In this case, the patient presented with classic symptoms of dengue fever, which led clinicians to maintain a low threshold for suspicion. Initial laboratory investigations confirmed severe thrombocytopenia with a platelet count of 25 × 10^9^/L. Although a subsequent platelet count showed a slight improvement to 40 × 10^9^/L, the value remained significantly low. Nevertheless, the patient remained clinically stable throughout the evaluation.

In a case reported from Brazil, a breast cancer patient undergoing chemotherapy presented with a platelet count of 19 × 10^9^/L in an EDTA-anticoagulated sample. Similar to our case, the peripheral blood smear did not reveal true thrombocytopenia, and both white and red blood cell counts were within normal limits. The patient’s chemotherapy was delayed due to the presumed diagnosis of chemotherapy-induced thrombocytopenia. This case highlights how EDTA-PTCP can alter clinical decision-making and emphasizes the need to raise awareness of this phenomenon to avoid unnecessary delays and complications[15].

A similar case was reported from India involving a child with confirmed dengue fever and co-existing EDTA-PTCP. The platelet count was markedly low at 5 × 10^9^/L in the EDTA sample but significantly higher at 85 × 10^9^/L in samples collected using heparin and citrate tubes. While this case differs from ours in that EDTA-PTCP amplifies a true underlying thrombocytopenia rather than creating a false one, it reinforces the same critical point: EDTA-PTCP can substantially distort the true platelet count. This diagnostic interference can mislead clinicians regarding both the presence and degree of thrombocytopenia. Therefore, awareness and recognition of EDTA-PTCP remain essential in all clinical contexts where platelet counts guide management decisions[16].

In such scenarios, it is crucial for physicians to exercise caution before proceeding with invasive interventions, such as bone marrow biopsy, or administering unnecessary treatments, including platelet transfusions. While platelet transfusion is generally considered safe, it is not without risk. Potential complications include febrile non-hemolytic and anaphylactoid reactions, which are frequently associated with transfusion of platelet concentrates. More severe complications, including septic reactions due to bacterial contamination, transfusion-related acute lung injury, and life-threatening anaphylaxis, have been documented[17].

Therefore, in the evaluation of patients with severe thrombocytopenia that does not correlate with the clinical presentation, standard hematological testing methods can aid in the accurate detection of EDTA-PTCP. A peripheral blood smear, considered the gold standard for detecting EDTA-PTCP, should be performed. If platelet clumping is observed, a repeat sample should be collected using an alternative anticoagulant. Sodium citrate, heparin, magnesium sulfate, or EDTA mixed with amikacin may be used to confirm the true platelet count^[18,19]^. Additionally, manual platelet counting by microscopy is recommended for more accurate assessment.

## Conclusion

In regions where dengue fever is prevalent, maintaining a high index of suspicion is essential; however, clinicians must also consider EDTA-PTCP as a differential diagnosis in cases of thrombocytopenia. Peripheral blood smear examination should be included in the diagnostic workup of thrombocytopenia to verify platelet counts and rule out clumping. Clinical context remains pivotal in guiding appropriate management and in preventing unwarranted interventions. Close monitoring of the patient’s symptoms and bleeding tendency is imperative. Additionally, a thorough understanding of the various types of blood collection tubes, including their specific indications and limitations, can enhance diagnostic accuracy and clinical judgment.

## Data Availability

I confirm that any datasets generated during and/or analyzed during the current study are available upon reasonable request.

## Research registration unique identifying number (UIN)

Name of the registry: N/A.

Unique Identifying number or registration ID: N/A.

Hyperlink to your specific registration (must be publicly accessible and will be checked): N/A.
